# Non-Linear Resistance Training Program Induced Power and Strength but Not Linear Sprint Velocity and Agility Gains in Young Soccer Players

**DOI:** 10.3390/sports6020043

**Published:** 2018-05-14

**Authors:** Matheus Barbalho, Paulo Gentil, Rodolfo Raiol, Fabrício Boscolo Del Vecchio, Rodrigo Ramirez-Campillo, Victor Silveira Coswig

**Affiliations:** 1 Centro de Ciências Biológicas e da Saúde, Universidade da Amazônia, Belém 66035-190, Brazil; 2 Faculdade de Educação Física e Dança, Universidade Federal de Goiás, Goiania 74690-900, Brazil; paulogentil@hotmail.com; 3 Centro de Ciências Biológicas e da Saúde, Centro Universitário do Estado do Pará, Belém 66040-020, Brazil; rodolforaiol@gmail.com; 4 Faculdade de Educação Física, Universidade Federal de Pelotas, Pelotas, Rio Grande do Sul 96055-630, Brazil; fabricioboscolo@gmail.com; 5 Department of Physical Activity Sciences, Research Nucleus in Health, Physical Activity and Sport, Universidad de Los Lagos, Osorno 1305, Chile; r.ramirez@ulagos.cl; 6 Faculdade de Educação Física, Universidade Federal do Pará, Castanhal 68746-630, Brazil; vcoswig@gmail.com

**Keywords:** soccer, non-linear periodization, strength training, young players

## Abstract

**Background:** The present study evaluated the effects of resistance training (RT) following a non-linear periodization model in the physical fitness of young soccer athletes. **Methods:** Young soccer players (*n* = 23) were allocated into two groups: an RT group (RTG), and the control group (CON). The RTG underwent 15 weeks of non-linear RT periodization in three weekly sessions in addition to their specific soccer training. The CON continued performing the specific soccer training. Before and after the training period, all of the subjects performed one-repetition maximum (RM) tests for speed, agility, and power (vertical and horizontal jump). **Results:** The RTG obtained significant gains in one-RM tests (before 64.1 ± 5.8 kg, after 79.1 ± 3.3 kg) and power (vertical jump (before 56 ± 2.7 cm, after 61.3 ± 1.7 cm) and horizontal jump (before 184.5 ± 5.5 cm, after 213.6 ± 3.2 cm)). In contrast, the CON group presented a non-significant increase in one-RM tests and horizontal jump, and a significant reduction in vertical jump (before 55.4 ± 2.2 cm, after 51.3 ± 1.5 cm). Neither group presented significant gains in speed (CON: *p* = 0.27; RTG: *p* = 0.72) and agility (CON: *p* = 0.19; RTG: *p* = 0.58). **Conclusion:** Our data suggest that non-linear RT should be inserted into the routine of young soccer athletes for improving strength and power without impairing speed and agility.

## 1. Introduction

The development of competitiveness in soccer requires a high improvement of physical conditioning, especially strength, power, and speed [1]. In a match, it is estimated that the average athlete performs from 1000 to 1400 movements, in which 150 to 250 are of high intensity, depending on age and position [1]. It has been suggested that muscular power is a determining factor in soccer, either in high-speed runs [2], vertical jumps to head a ball [3], or throws in the lateral charges and ball replacements of goalkeepers [4]. More than that, muscular power measured by sprinting or jumping seems to be strongly related to change of direction and agility [5]. Such high physical demand rises the need for programs that develop strength, power, agility, and speed [6,7,8]. In addition, linear sprint velocity and power are key components of soccer-specific physical conditioning, which include acceleration, repeated anaerobic sprint ability, and lower extremity explosive power [9]. These suggests that strength, speed, and endurance are important while attacking and defending in soccer [10]. Resistance training (RT) has been considered fundamental for the development of strength, power, speed, and endurance, and is used in several high-performance sports [11,12], including soccer [2,13]. However, it is worth emphasizing that each prescription must respect the specificity of modalities and their athletes, in order to optimize the results from the RT [14].

In this sense, a previous study compared the performance of young athletes performing strength training and soccer-specific aerobic training. In two years, the group that performed RT showed higher increases in speed and maximum strength [2]. A study that measured the neuromuscular fitness of lower limbs in young athletes found that the performance of RT resulted in significant gains in isokinetic, power, and velocity tests, which did not occur with the control group [15]. Subsequently, the same group analyzed kicking power in young athletes, and the group that performed RT showed gains in kick power and maximum strength, while the control group did not [16].

In general, physical conditioning with programs of strength, power, and speed are elaborated, considering different models of periodization, which aim to enhance these physical abilities [17]. RT programs are frequently organized according to the linear periodization system, where changes in volume and intensity are performed at each mesocycle [18] differently from non-linear periodization where changes in volume and intensity occur daily or weekly [19].

Although the use of periodization in sports training is widely discussed [17,20], studies have shown that non-linear periodization could be a more interesting strategy than traditional strategy, both in trained and untrained men [18,21]. One possible advantage of non-linear periodization for the modern competitive schedule is that athletes compete at a high frequency (many times per month, in the case of soccer); therefore, providing different training stimuli over a short time period might be beneficial, especially for those activities that involve many physical capacities. In the sports environment, the use of non-linear periodization was not often utilized [18,19,22]. Specifically in relation to soccer, no studies were found that applied non-linear periodization.

The present study aimed to analyze the use of RT following non-linear periodization in relation to gains of muscular strength, speed, agility, and power during 15 weeks of RT in players of the U-20 soccer category. Considering that the use of non-linear periodization has been shown to promote positive results in strength gains [21], power [22], and velocity [23] in other groups of athletes and non-athletes, the hypothesis of the present study is that the addition of RT, following a non-linear periodization model, would promote performance improvements in young soccer players in comparison to specific soccer training alone.

## 2. Materials and Methods

### 2.1. Experimental Approach to Problem

After two initial weeks of evaluation, participants were randomly assigned to two groups by block randomization [24]: (1) a group that performed RT following non-linear periodization in addition to soccer training (RTG, *n* = 11); and (2) a group that performed only conventional soccer training (CON, *n* = 12). The two initial weeks involved familiarization with RT, anthropometric evaluation, performance of the tests, and re-tests of maximum strength, speed, agility, and power. During the familiarization sessions, the participants were instructed on the correct execution of the exercises. The initial values of the maximum strength test (repetition maximum, or RM) in the squat were obtained, as well as the speed, agility, and power tests: 40-yard sprint, T-test, and vertical and horizontal jump [25].

### 2.2. Participants

A priori sample size test (G*Power 3.0.10) was applied, considering countermovement jump (CMJ) responses (effect size—ES = 1.33) found in a previous study with young soccer players [26]. Based on an estimated power of 0.9 and a level of significance of 5% in a two-sided paired T-test, at least nine subjects would be required for each group. Therefore, 23 U-20 soccer athletes from a professional club, with no RT experience, were enrolled in the present investigation. The inclusion criteria was: to be between 18–20 years old, to have been practicing soccer for more than 12 months, and to have a weekly practice frequency of more than 80%. Athletes with orthopedic lesions or cardiological alterations that could be aggravated by the study protocol were excluded. Participants were asked not to change their nutritional habits during the study, and did not use supplements or medications to improve the performance. All of the participants were previously notified about the experimental procedures, benefits, and risks of the study, and it was required for all of the participants to sign a consent form. The study followed the principles of the Declaration of Helsinki, and was approved in the local committee of ethics in research with human beings (protocol number 005/2012).

A random allocation was made by simple draw considering the two groups, which occurred in the period of evaluations and initial tests. The professionals who followed the sessions were also randomized and distributed on work scales by simple draw, to be certain that the same number of sessions for each group would be applied. Although it was impossible to blind the participants and supervisors to the intervention groups, tests and data typing were performed by researchers who were blinded to group allocation.

### 2.3. Assessments

#### 2.3.1. Anthropometric Measures

Body mass was measured using a scale with 50-g precision. Height was measured with a wall-mounted stadiometer with an accuracy of one millimeter (Filizola PL200). Body mass index was calculated using body mass in kilograms and height in meters squared [27].

#### 2.3.2. One Repetition Maximum Test (1 RM)

One week before the tests, the participants conducted two training sessions for familiarization. During the familiarization sessions, each exercise was performed with a self-selected load that would comfortably allow the performance of 15 repetitions in two consecutive sets. One week prior to the start of the training period and five to seven days after the last session, maximum strength was measured by the one-RM test for the squat exercise (LonglifePro brand, line LLP-NE200, Maringá, Brazil) using Olympic bars (Auriflama, Brazil) and plate loads from 0.5 to 20 kg. On the test day, participants performed a warm-up consisting of eight repetitions at 40 to 50% of their estimated one RM. After a rest interval of 60 s, they performed six repetitions at 50 to 60% of their estimated one RM. Then, the load was increased and an attempt was made. If the repetition was successfully completed, the load was increased between 1–2 kg and, after a five-minute interval, another attempt was performed. The procedure was repeated until achieving the highest load with which the individual could perform a complete repetition. A maximum of five attempts per session was allowed. If the maximum load was not obtained by the fifth attempt, the test was interrupted and repeated after 48 h; however, this was not necessary for any of the participants. The full range of motion was the point where the thighs were parallel to the ground. Subjects received verbal stimulation throughout the test, and the same group of researchers, blinded to group allocation, performed all of the procedures. The re-test was performed the same way 48 h to 72 h after the test and resulted in an intraclass correlation coefficient (ICC) > 0.99 [28].

#### 2.3.3. Speed

The speed test used was the 40-yard sprint. It is commonly used for modalities in which extensive runs are performed, such as soccer, American football, rugby, and field hockey [26]. The test consisted in the athlete positioning behind a starting line and waiting for a countdown to begin the test. Verbal stimulus was used to notify the beginning of the test, and a hand-held chronometer was used to measure time. This test presents ICC > 0.98 and 95% CI when compared with electronic timers [29].

#### 2.3.4. Agility

The T-test is one of the most common tests of agility, and is widely used in sports such as soccer, basketball, and baseball, all of which require speed with fast forward, lateral, and backward movement changes [30]. The test initially consisted of positioning four cones, the first cone being the starting line for the test, and the second cone being positioned 9 m from the first. Then, two cones are positioned at 4.5 m on the right side and left side of the second cone. In this way, the athlete begins the test by running 9 m straight and touching the base of the second cone with the right hand. The athlete then runs 4.5 m to the left and touches the base of the third cone with the left hand. Then, the athlete runs 9 m from left to right, to the fourth cone, and touches the base of the cone with his right hand. Finally, the volunteer runs sideways to the second cone, touches it with his left hand, and then runs back to the initial cone [31]. Time was measured using a hand-held chronometer. This test presents an ICC > 0.98 and 95% CI when compared with electronic timers [29].

#### 2.3.5. Muscle Power

Vertical jumping is one of the most used power tests in strength and conditioning due to the ease of administering the test, but also because the results are directly applicable to most sports that require the explosive movements of lower limbs [32]. Initially, the test consists of measuring the height of the volunteer, with the dominant arm raised as high as possible [33]. Then, the participant jumps as high as possible, and the farthest point he reaches in relation to the ground is marked [33]. The height of the jump is recorded as the difference between the two marks [34]. The horizontal jump test is another test that is used for the performance of lower limb power. The test consists of an initial warm-up of five minutes of self-selected moderate intensity aerobic exercises, then ballistic exercises of hip flexion and extension. The test started with both feet positioned before a demarcated line, and the participant jumped as far as possible. Both tests were repeated three times, with an interval of two minutes between attempts, and the best performance of each type of jump is recorded [34]. This test shows an ICC value of 0.99 and 95% CI in young soccer players.

#### 2.3.6. Specific Training

Both groups performed aerobic training, using intensities from 60% to 80% and between 80–95% of maximal heart rate (Speedo^®^ Heart Rate Monitor, São Paulo, Brazil), with variations throughout the training process. The tactical training was at the discretion of the technical committee, and was coordinated by the physical trainer. These stimuli were performed five times a week, at a different shift from the strength training of the experimental group, with a total time of 40 min to 80 min per session. The participants were from the same team; therefore, specific training was identical for both groups.

#### 2.3.7. Strength Training Protocol

Strength training was performed three times a week. One day for upper limbs (bench press, lateral pull down, shoulder press, seated low row, triceps pulley, and biceps curl) and two for lower limbs (leg press 45°, free squatting with bar, seated leg curl, calf standing in the machine). Each session was separated by at least 48 h (Table 1). The exercises were performed with three sets for multi-joint exercises, and two series for single joint exercises.

All of the participants were supervised and monitored in all of the exercises, since this has been shown to influence the results [35,36]. The training followed a model of non-linear periodization. During weeks 1, 5, 9, and 13, participants performed 12–15 RM with 30–60 s intervals between sets. During weeks 2, 6, and 10 and 14, 4–6 RM were performed with 3–4 min intervals. Weeks 3, 7, 11, and 15 involved 10–12 RM with 1–2 min intervals. During weeks 4, 8, and 12 the participants performed 6–8 RM at 2–3 minute intervals (Table 1). Participants were instructed to perform each series until voluntary failure [37], as previously defined, and in case they could perform more repetitions than indicated, a load of 2.5 to 5 kg was added for the next training session. The participants were instructed to perform the concentric and eccentric phases in two seconds each, without pause between phases.

### 2.4. Statistical Analysis

After verification of the normality of the data by the Shapiro–Wilk’s test and of the equality of variances by Levene’s test, all of the variables showed normal distribution and were reported by means ± standard deviation. Within-group comparisons (PRE and POST) were performed by the paired Student’s *t*-test, and an analysis of covariance (ANCOVA) was used to compare the absolute change in each outcome variable between groups with pre-test scores used as a covariate, and 95% confidence intervals (CI) were examined for within-group change. Significant within-group change was considered to have occurred if the 95% CIs for changes did not cross zero. ES was calculated as proposed by Cohen [38]. Statistical analysis was performed using JASP (version 0.8.1.2; University of Amsterdam, Amsterdam, The Netherlands), with alpha for significance accepted at ≤0.05.

## 3. Results

The CON group (*n* = 11) had a mean age of 19.1 ± 0.9 years, height of 176.33 ± 8.57 cm, and body mass of 72 ± 5.9 kg. The RTG group had a mean age of 18.8 ± 0.8 years, height of 178.4 ± 6.2 cm, and body mass of 73.1 ± 6.6 kg. No significant differences between groups were identified for these parameters.

Table 2 describes the results of maximum strength (one RM), muscle power (vertical jump and horizontal jump), speed (40-yard sprint) and agility (T-test). The RTG group showed increases that were statistically significant on the one-RM test (64.1 ± 5.8 kg versus 79.1 ± 3.3 kg), in vertical jump height (56 ± 2.7 cm versus 61.3 ± 1.7 cm), and in horizontal jump distance (184.5 ± 5.5 cm versus 213.6 ± 3.2 cm). In contrast, the CON group presented a non-significant increase in the one-RM test (63.3 ± 5.3 kg versus 66.2 ± 5.2 kg), a non-significant reduction in horizontal jump distance (181.3 ± 4 cm versus 180.1 ± 3.9 cm) and a significant reduction in vertical jump height (55.4 ± 2.2 cm versus 51.3 ± 1.5 cm). The 40-yard sprint time and T-test did not show significant differences within or between groups.

The ANCOVA results are shown in Table 3, and indicate that the RTG presented greater changes in the squat one-RM test (F = 138.4, *p* < 0.001; ES = 1.92), vertical jump height (F = 190.4, *p* < 0.001; ES = 4.46), and horizontal jump height (F = 1537.5, *p* < 0.001; ES = 5.02) when compared with the CON. There were no significant differences in the 40-yard sprint test (F = 2.9, *p* = 0.10, ES = 0.23) and T-test (F = 1.8, *p* = 0.19, ES = 0.31) between groups.

## 4. Discussion

The present study aimed to investigate the effects of 15 weeks of RT on the muscle strength, speed, agility, and power of soccer players of the U-20 category. The main findings were the gains in muscular strength and power only for the RTG, with no effect on speed and agility tests. These results confirm the initial hypothesis for non-linear periodization RT improvement in muscle strength and muscle power, while the hypothesis for speed and agility gains were not confirmed.

Regarding muscle strength, Sander et al. [2] followed two groups of young soccer players in the U13, U15, and U17 categories for two years. One group performed RT two times a week, in addition to conventional soccer training, while the other only performed soccer-specific physical training. Corroborating with the present study, the results showed that the one-RM tests increased only with RT. In a similar study, Di Giminiani and Visca [26] investigated the effects of RT in young soccer athletes for two seasons, and found strength gains of 10.3% in 19 athletes with a mean age of 13 years old. Barjaste and Mirzaei [39] also found similar results when comparing two groups of young soccer players, one of whom performed RT in a linear fashion, and the other who did not perform RT. The results showed gains of 29.3% and 9.6% in the adaptation (65% and 75% of one RM) and maximal strength phases (85% to 95% of one RM), respectively. The novelty of the present study is the use of non-linear periodization. By doing this, we confirm that, independent of the model used, RT is able to increase muscle strength when compared with soccer-specific training [8].

Power gains were evidenced by Di Giminiani and Visca [26] in adolescent soccer players submitted to RT (7.6%) when compared with adolescent soccer players who did not practice RT. Similar results were found by Chelly et al. [7], in which the researchers compared two groups of young soccer players, one group performing RT twice weekly with loads of 70%, 80% and 90% of one RM, and one control group who did not perform RT. The intervention lasted two months, and the group that performed RT showed significant gains in peak power when compared with the group that did not perform RT. These results reinforce the recommendation for the inclusion of RT for power gains in young soccer players, since this physical capacity has a high relevance in the modality and presents as one of the determinants for competitive success, such as sprints, kicking, and jumping [3,4]. Notwithstanding, improvements found in the horizontal (29%) and vertical jumps (5.4%) were not followed by significant changes in the linear sprint velocity. This could be explained by a limitation in our tests, since we were not able to estimate the previously mentioned relative to body mass (N·kg^−1^), which is more related to sprint performance [40].

Regarding speed and agility gains, Sander et al. [2] found higher gains in the group that practiced RT compared with the group that did not practice RT. The intervention lasted two years and showed superiority in the speed gains for the RT group, especially in short distances (5 m and 15 m), which may be related to the increase of the muscular strength in the group that was submitted to the RT. Additionally, linear sprint velocity gains were evident in young soccer players performing RT, either with linear models [7,17] or based on the optimal power load [17]. In fact, these findings differ from the present study, in which the RT did not induce significant improvements in speed and agility in the young soccer players. These differences can be justified by three aspects: (i) higher exposure time at high loads (80–90% one RM) in a non-linear model [7]; (ii) the addition of specific power drills such as squat jumps [17]; and/or (iii) a suggested weak relationship between agility and strength [5]. Thus, the authors suggest that these particularities should be considered for RT programming when the purpose is to induce gains in speed and agility.

To the authors’ knowledge, the present study was the first to propose the application of RT following a non-linear periodization model in young soccer players. Positive results for strength and power were obtained; however, there were no gains in speed and agility. Perhaps the repetition variations and intensity ranges [19] might not be indicated for speed and agility gains because it does not allow enough exposure to the specific power drills that are important for the improvement of these variables [17]. Therefore, the present study suggests the non-linear periodization to be adapted with the inclusion of power drills along with strength, hypertrophy, and RT to increase gains related to agility and speed.

The main limitation of this study is the lack of a cross-over design; however, the authors’ choice of design was based on the objective of investigating the responses to non-linear periodization with sufficient duration to increase the consistency and practical application of the findings, since logistically it was necessary to choose between longer-term follow-up or cross-delineation with short-term effects. On the other hand, one of the strengths of the present study is the application of a model that had not yet been tested in this population. Thus, the model of non-linear periodization may be an alternative strategy to classic periodization models, especially regarding amateur soccer and school environments, where competitions occur throughout the year, and it is difficult to establish peak training phases. However, it is important to reinforce the need for adaptations for increasing exposure to higher loads and/or for the insertion of additional specific methods for speed and agility enhancement.

## 5. Conclusions

Our findings suggest that RT following a non-linear periodization model increased the performance of young soccer players in one-RM tests and horizontal and vertical jumps, but not in linear sprint velocity and agility. Therefore, we conclude that this model might be an interesting strategy for increasing soccer athletes’ strength and power, while the addition of specific exercises is suggested for speed and agility improvements. Finally, practical application of this model could be related to the maintenance of physical fitness when coaches may not have specific dates for peak performance.

## Figures and Tables

**Table 1:** Training division and protocol.

Training A		Training B	
Leg Press 45°		Flat Bench Press	
Back Barbell Squat		Lateral Pull Down	
Seated Leg Curl		Military Press	
Calf Raise		Seated Cable Low Row	
		Triceps Pulley	
		Biceps Curl	
Weeks	Repetitions/Load		Rest
1, 5, 9, and 13	12–15 RM		30–60 s
2, 6, 10, and 14	4–6 RM		3–4 min
3, 7, 11, and 15	10–12 RM		1–2 min
4, 8, 12	6–8 RM		2–3 min

**Table 2:** Characteristics of the participants before (Pre) and after (Post) the training period (mean ± standard deviation). CON: control group, RTG: resistance training group, RM: repetition maximum.

	CON (*n* = 11)			RTG (*n* = 12)		
	Pre	Post	*p*	Pre	Post	*p*
One RM (kg)	63.3 ± 5.3	66.2 ± 5.2	0.203	64.1 ± 5.8	79.1 ± 3.3	<0.001
Sprint 40-yard (s)	5.4 ± 0.5	5.7 ± 0.4	0.277	5.4 ± 0.5	5.5 ± 0.5	0.724
T-test (s)	11.6 ± 0.6	12.1 ± 0.8	0.192	11.5 ± 0.6	11.7 ± 0.6	0.588
Vertical Jump (cm)	55.4 ± 2.2	51.3 ± 1.5	<0.001	56 ± 2.7	61.3 ± 1.7	<0.001
Horizontal Jump (cm)	181.3 ± 4	180.1 ± 3.9	0.429	184.5 ± 5.5	213.6 ± 3.2	<0.001

**Table 3:** Change in outcomes over the training period (marginal mean ± standard error) in addition to 95% CIs.

	CON (*n* = 11)		RTG (*n* = 12)			
	Change	95% CI	Change	95% CI	F	*p*
One RM (kg)	2.6 ± 6.4	−1.6 to 6.9	14.9 ± 6.2	10.7 to 19.0	138.4	<0.001
Sprint 40-yard (s)	0.3 ± 0.8	−0.2 to 0.8	0.1 ± 0.8	−0.4 to 0.6	2.9	0.108
T-test (s)	0.5 ± 1.3	−0.3 to 1.4	0.2 ± 1.1	−0.5 to 0.9	1.8	0.192
Vertical Jump (cm)	−4.1 ± 1.8	−5.2 to −2.9	5.4 ± 2.4	3.7 to 6.9	190.4	<0.001
Horizontal Jump (cm)	−1.2 ± 4.8	−4.3 to 2.0	29.1 ± 6.6	24.6 to 36.5	1537.5	<0.001
